# Water-clear cell parathyroid adenoma: A case report and mini-review of the literature

**DOI:** 10.3892/mi.2025.269

**Published:** 2025-09-17

**Authors:** Ari M. Abdullah, Aras J. Qaradakhy, Rebaz M. Ali, Abdulwahid M. Salih, Hiwa O. Baba, Rebaz O. Mohammed, Abdullah A. Qadir, Hawkar A. Nasralla, Shko H. Hassan, Fahmi H. Kakamad

**Affiliations:** 1 Department of Scientific Affairs, Smart Health Tower, Sulaymaniyah 46001, Iraq; 2 Department of Pathology, Sulaymaniyah Teaching Hospital, Sulaymaniyah 46001, Iraq; 3 Department of Radiology, Shorsh Teaching Hospital, Sulaymaniyah 46001, Iraq; 4 Department of Oncology, Hiwa Cancer Hospital, Sulaymaniyah 46001, Iraq; 5 Zad Organization, Sulaymaniyah 46001, Iraq; 6 College of Medicine, University of Sulaimani, Sulaymaniyah 46001, Iraq; 7 Kscien Organization for Scientific Research (Middle East Office), Sulaymaniyah 46001, Iraq

**Keywords:** water-clear cell parathyroid adenoma, hyperparathyroidism, parathyroidectomy, rare parathyroid neoplasm, histopathological diagnosis

## Abstract

Water-clear cell parathyroid adenoma (WCCPA) is a rare variant of parathyroid adenoma, characterized by large polygonal cells with optically clear cytoplasm. To date, only a limited number of cases have been reported in the English literature. The present study describes the case of a patient with WCCPA. An 80-year-old female presented with muscle pain and fatigue lasting for 1 week. A clinical examination revealed a normal thyroid gland with no palpable masses. Laboratory tests revealed elevated levels of serum calcium (12.73 mg/dl) and parathyroid hormone (PTH; 303 pg/ml). A neck ultrasonography revealed a 28x16x11 mm hypoechoic, mildly vascular lesion, suspicious of a parathyroid nodule, located posterior to the left thyroid lobe. A left lower parathyroidectomy was performed, and a histopathological examination confirmed the diagnosis of WCCPA. Post-operatively, the serum calcium and PTH levels of the patient normalized, and her recovery was uneventful. In addition, a review of 4 recent cases of WCCPA found in the literature demonstrated a common clinical pattern of elevated calcium levels and increased PTH levels. Imaging techniques, such as ultrasonography and sestamibi scans revealed localized parathyroid lesions, which were subsequently confirmed as WCCPA through histopathological evaluation. Surgical excision was performed in all cases, resulting in postoperative normalization of calcium levels in the majority of patients. WCCPA is a rare, yet significant cause of primary hyperparathyroidism. An accurate diagnosis relies on a histopathological examination, as clinical and imaging findings may be non-specific. Surgery can yield favorable outcomes.

## Introduction

Water-clear cell parathyroid adenoma (WCCPA) is an extremely rare type of parathyroid neoplasm characterized by large polygonal cells with an optically clear cytoplasm (1,2). This rare variant accounts for <1% of all primary hyperparathyroidism cases, with only a limited number of cases reported in the English literature (1,3). WCCPA predominantly affects middle-aged females, although cases in both sexes and varying ages have been observed (2). These tumors tend to present with non-specific symptoms, including bone pain, nephrolithiasis, or hypercalcemia-related signs, and can remain asymptomatic until the adenoma becomes large enough to cause compressive symptoms (1). Despite its rarity, WCCPA can be challenging to diagnose pre-operatively due to inconsistent uptake on imaging analyses, such as sestamibi scans and ultrasound, frequently resulting in false-negative findings (3). The diagnosis is typically confirmed post-operatively through a histopathological examination (HPE), revealing vacuolated clear cells within the parathyroid tissue (4). Surgical resection remains the primary treatment strategy, and outcomes are generally favorable, with the majority of patients achieving normocalcemia post-operatively (1,2).

The present study describes the case of a patient with WCCPA and provides a brief literature review in an aim to enhance the understanding of this rare entity. All referenced sources were evaluated for reliability, and the report has been prepared in accordance with the CaReL guidelines (5,6).

## Case report

*Patient information*. An 80-year-old female presented to Smart Health Tower, Sulaymaniyah, Iraq, with muscle pain and fatigue lasting for 1 week. No notable findings were identified in her medical and surgical history.

### Clinical findings

During a clinical examination, no thyroid nodules or lymphadenopathy were detected.

### Diagnostic assessment

Routine laboratory tests, including a complete blood count (CBC) with red blood cell, white blood cell and platelet measurements, were all within normal limits. The analysis of CBC was performed using a Swelab Alfa Plus analyzer (Boule Medical AB). The parathyroid hormone (PTH) level was 303 pg/ml (reference range, 10-65 pg/ml), measured using electrochemiluminescence immunoassay (ECLIA) on a Roche Cobas pro e801 analyzer (Roche Diagnostics GmbH). The serum calcium level was 12.73 mg/dl (reference range, 8.5-10.5 mg/dl), measured using a colorimetric method on a Roche Cobas pro C503 analyzer (Roche Diagnostics GmbH). A neck ultrasonography revealed thyroid lobes of normal size with homogeneous echotexture and a few small TR2 and TR3 nodules (<7 mm in size) exhibiting normal vascularity (images not available). No significant cervical pathological lymphadenopathy was observed. A solid, hypoechoic, mildly vascular nodule (suspicious for parathyroid lesion) measuring 28x1,611 mm was identified posterior to the lower third of the left thyroid lobe, extending retrosternally, with normal surrounding tissue, suggestive of a parathyroid nodule.

### Therapeutic intervention

**Figure 1:**
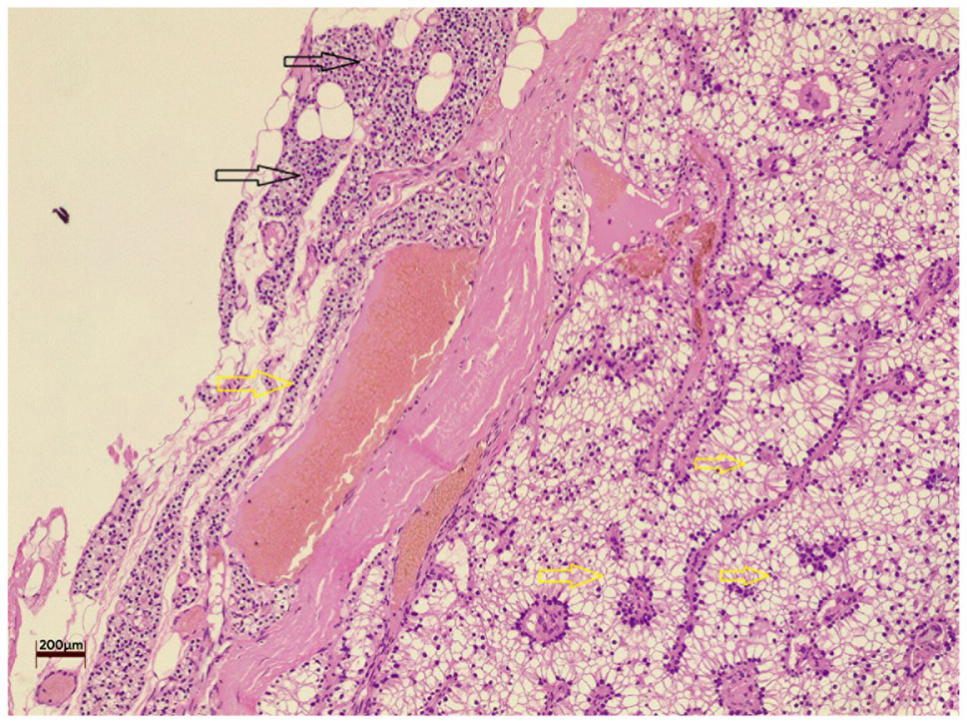
Section illustrating a well-circumscribed mass composed of cells with uniform nuclei and clear cytoplasm (yellow arrows), adjacent to a small, unremarkable area of normal parathyroid tissue (black arrows). Hematoxylin and eosin staining; magnification, x10; scale bar, 200 µm.

A left lower parathyroidectomy was performed under general anesthesia with the patient in the supine position. The excised specimen was sent for a histopathological examination. Histological analysis was performed on 5-µm-thick paraffin-embedded sections. The sections were fixed with 10% neutral buffered formalin at room temperature for 24 h and then stained with hematoxylin and eosin (Bio Optica Co., Italy) for 1-2 min at room temperature. The sections were then observed under a light microscope (Leica Microsystems GmbH, Germany). The histological analysis revealed a WCCPA (Fig. 1).

### Follow-up and outcome

The patient was discharged on the first post-operative day following an uneventful recovery. The post-operative PTH level was 42.4 pg/ml, and serum calcium levels had normalized to 9.31 mg/dl.

## Discussion

In the present study, four recently reported cases of WCCPA were reviewed. The ages of the patients ranged from 56 to 70 years, with 2 males and 2 females; 3 cases were reported from the USA and 1 case was from Taiwan. Past medical histories included conditions, such as osteoporosis, hypertension and Barrett's esophagus, and presentations commonly involved hypercalcemia and bone pain. Laboratory findings revealed elevated calcium and PTH levels, and imaging via ultrasonography and computed tomography (CT), and Tc-99m sestamibi scans revealed parathyroid masses. Surgical management, primarily parathyroidectomy and lesion resection, was the treatment of choice. Post-operatively, the majority of patients achieved the normalization of PTH and serum calcium levels. Histopathological examinations confirmed the diagnosis of WCCPA in all cases. Clinical outcomes were generally favorable, with patients either remaining asymptomatic or demonstrating significant improvement (1-4) (Table I).

WCCPA is characterized by a unique water-clear cytoplasmic appearance, attributed to the accumulation of glycogen and intracellular fluid. These clear cells are considered to stem from chief cells that undergo cytoplasmic changes rather than representing a separate cellular origin. Despite their rarity, these tumors are important to recognize due to their association with primary hyperparathyroidism, often presenting with elevated calcium and PTH levels (1). WCCPA typically affects patients in their sixth or seventh decade of life, with a slight female predominance, although the review of cases performed herein revealed a male-to-female ratio of 1:1, slightly differing from the general trend (2,3). The clinical presentation of WCCPA largely mirrors that of primary hyperparathyroidism, with patients frequently exhibiting signs and symptoms of hypercalcemia, such as fatigue, bone pain and muscle weakness. Nevertheless, some individuals remain asymptomatic, and the condition is discovered incidentally through routine biochemical screening (1-4). In line with this variability, the case in the present study was an 80-year-old female who presented with a 1-week history of muscle pain and fatigue, ultimately leading to the diagnosis of WCCPA.

Although elevated serum calcium and PTH levels are characteristic of WCCPA, they are not specific to this entity. These biochemical abnormalities are shared with several other conditions, including other parathyroid adenomas, parathyroid carcinoma, tertiary hyperparathyroidism, familial hypocalciuric hypercalcemia and hypercalcemia of malignancy (7). Therefore, a laboratory evaluation serves primarily as a preliminary screening method. Distinguishing WCCPA from these entities requires a combination of clinical assessment, targeted imaging modalities and a histopathological examination supported by immunohistochemical staining to exclude morphologically similar lesions. Herein, in the reviewed cases, all patients exhibited hypercalcemia, which is the hallmark of primary hyperparathyroidism, along with elevated levels of PTH. Serum calcium levels ranged from mildly elevated to significantly high values, often exceeding 12 mg/dl, a level commonly associated with hyperparathyroidism (1,2). As in the present case report, the PTH level was 303 pg/ml and the serum calcium level was 12.73 mg/dl.

The diagnosis of WCCPA requires a high index of suspicion, particularly in patients with atypical clinical presentations. While ultrasonography, CT and Tc-99m sestamibi scans are the most common imaging methods, they frequently only suggest a parathyroid adenoma without providing a definitive diagnosis of WCCPA. The majority of the reviewed cases in this report underwent neck ultrasonography and sestamibi scanning, which localized the hyperfunctioning parathyroid glands and confirmed WCCPA by histopathological analysis. Histologically, WCCPA is defined by sheets or lobules of water-clear cells with distinct cell borders, necessitating immunohistochemical staining to differentiate it from other clear cell neoplasms such as metastatic renal cell carcinoma or clear cell variants of thyroid carcinoma (4). In the present case, neck ultrasonography revealed a solid, hypoechoic, mildly vascular parathyroid nodule measuring 28x16x11 mm, located posterior to the lower third of the left thyroid lobe and extending retrosternally. The lesion was surrounded by normal tissue and was suggestive of a parathyroid nodule.

Surgical excision remains the treatment of choice for WCCPA, as medical management alone is insufficient to control hyperparathyroidism in these patients. Parathyroidectomy normalizes serum calcium and PTH levels, alleviating hypercalcemia-related symptoms. All the cases reviewed herein underwent successful surgical resection, resulting in favorable outcomes with normalization of calcium and PTH levels post-operatively. Long-term follow-up is critical, as recurrences of WCCPA have been reported, albeit rarely. Although WCCPA is generally regarded as a benign neoplasm, there is the potential for local recurrence and, in extremely rare cases, metastasis (4,8,9). Due to the rarity of this tumor, specific data on long-term outcomes are limited; therefore, post-operative surveillance is necessary to monitor for recurrent hyperparathyroidism, particularly given the possibility of incomplete excision and the known recurrence risks associated with parathyroid adenomas (1-4). In the case present herein, a left-lower parathyroidectomy was performed to rule out the differential diagnosis of parathyroid pathologies. A diagnosis of WCCPA was made after removing the nodule.

A limitation of the present case report is that the diagnostic workup relied on serum calcium and PTH measurements, together with ultrasonography, none of which are sufficient to establish a definitive diagnosis of WCCPA. As discussed above, these findings are non-specific and may be observed in various other parathyroid and non-parathyroid conditions. While imaging analyses can help localize a suspected adenoma, they cannot differentiate WCCPA from other histological subtypes. Therefore, as in all previously reported cases, the definitive diagnosis in the present case was only achieved through a histopathological examination.

In conclusion, WCCPA is a rare, yet significant cause of primary hyperparathyroidism. An accurate diagnosis relies on a histopathological examination, as clinical and imaging findings may be non-specific. Surgery can yield favorable outcomes.

## Figures and Tables

**Table I:** Summary of 4 cases of water-clear cell parathyroid adenoma reported in the literature.

First author/year of publication	Country	Age, years	Sex	PMH/PSH	Presentation	Lab	Imaging findings	Management	HPE	Post-operation imaging	Outcome	(Refs.)
Durant, 2023	USA	70	M	Osteoporosis, hypertension, Barrett's esophagus	Small bowel obstruction, hypercalcemia	Ca: 12.1 mg/dl, PTH: 119.3 pg/ml	US: 2.7x2.1 cm neck mass, sestamibi scan uptake	Parathyroidectomy	WCCPA, 3.2x1.5x0.6 cm	N/A	Normocalcemia, no complications	(1)
Yang, 2022	Taiwan	56	M	Hypertension, tophaceous gout	Weight loss, bone pain, fatigue, and kidney stones	Ca: 12.7 mg/dl, PTH: 936.94 pg/ml	US: bilateral extrathyroidal tumors, sestamibi-avid	Surgical resection of bilateral adenomas	Double WCCPA, 5.6x3.5x3.0 cm, 32 g	N/A	Normocalcemia	(2)
Abdelmasih, 2023	USA	63	F	Osteoporosis	Severe osteoporosis	Ca: 9.8 mg/dl, PTH: 122 pg/ml	CT: 8 mm mass near thyroid, US: inconclusive	Parathyroidectomy	WCCPA, histopathological confirmation	N/A	Improved PTH and calcium	(3)
Edwards, 2024	USA	61	F	Osteoporosis	Hypercalcemia, routine laboratory analyses	Ca: 12.3 mg/dl, PTH: 332 pg/ml, phosphorus: 2.1 mg/dl	Tc-99m sestamibi: hyperactive parathyroid neck mass, CT: Retrosternal extension	Surgical resection	WCCPA, 7.5x5.0x3.0 cm, 60 g	N/A	Asymptomatic, normalized PTH and calcium	(4)

M, male; F, female; PMH, past medical history; PSH, past surgical history; WCCPA, water-clear cell parathyroid adenoma; US, ultrasonography; CT, computed tomography; PTH, parathyroid hormone; Ca, calcium; HPE, histopathological examination; N/A, not available.

## Data Availability

The data generated in the present study may be requested from the corresponding author.
